# Development of Peptide Entry Inhibitors Targeting the Endosomal Receptor NPC1 Binding Site of *Orthoebolavirus*

**DOI:** 10.3390/pathogens15060640

**Published:** 2026-06-16

**Authors:** Leah Liu Wang, Kendra Alfson, J. J. Patten, Marc E. Mattix, Yenny Goez-Gazi, Sean N. Avedissian, Robert A. Davey, Ricardo Carrion, Shi-Hua Xiang

**Affiliations:** 1 School of Veterinary Medicine and Biomedical Sciences, Nebraska Center for Virology, University of Nebraska-Lincoln, Lincoln, NE 68588, USA; leahwang517@163.com; 2 Texas Biomedical Research Institute, 8715 W. Military Drive, San Antonio, TX 78227, USA; kalfson@txbiomed.org (K.A.); ygoez@txbiomed.org (Y.G.-G.); rcarrion@txbiomed.org (R.C.J.); 3 National Emerging Infectious Diseases Laboratories, Boston University, Boston, MA 02115, USA; jjpatten@g.ucla.edu (J.J.P.); radavey@bu.edu (R.A.D.); 4 Nonclinical Pathology Services, LLC, 5920 Clubhouse Pointe Dr., Medina, OH 44256, USA; rwpath@gmail.com; 5 College of Pharmacy, University of Nebraska Medical Center, Omaha, NE 68198, USA; sean.avedissian@unmc.edu

**Keywords:** Ebola virus disease (EVD), entry inhibitor, peptide, Niemann–Pick C1 (NPC1), receptor-binding site (RBS), *Orthoebolavirus zairense* (EBOV), *Orthoebolavirus bundibugyoense* (BDBV)

## Abstract

*Orthoebolavirus* causes severe Ebola virus disease (EVD) and deadly outbreaks in humans. This infection occurs through macropinocytosis and trafficking to late endosomes or lysosomes that utilize the receptor Niemann–Pick C1 (NPC1) to enter the cell cytoplasm. We designed peptide inhibitors based on the NPC1 receptor to target the NPC1 binding site to block viral entry. The results indicated that the ligand-based peptide inhibitors showed potent inhibition activities in vitro studies against pseudotyped or replication-competent *Orthoebolavirus*. Therefore, we further evaluated one of them in a mouse model challenged with mice-adapted Ebola viruses, which showed some protection efficacy compared with the control group. This study suggests that ligand-based peptides are encouraging inhibitors in the development of inhibitors against Ebola virus infection.

## 1. Introduction

*Orthoebolavirus* (old name *Ebolavirus*) is a genus of the family *Filoviridae*, according to the ICTV’s new classification in 2023, and has six recognized species, including *Orthoebolavirus zairense* (EBOV), *Orthoebolavirus bundibugyoense* (BDBV), *Orthoebolavirus sudanense* (SUDV), *Orthoebolavirus restonense* (RESTV), *Orthoebolavirus taiense* (TAFV), and *Orthoebolavirus bombaliense* (BOMV) [1,2]. *Orthoebolavirus* causes severe Ebola virus disease (EVD) and deadly outbreaks with mortality rates ranging from 25% to 90%, except for RESTV, which appears to be nonpathogenic [3,4]. Although EVD was discovered in Africa in 1976, there are still no effective drugs available, although two monoclonal antibody-based treatments (REGN-EB3 and mAb114) were approved in 2020 by the FDA [5]. These antibody-based drugs have several limitations, including being strain-specific, which possibly only works for EBOV but not for other strains such as BDBV that causes the current outbreak in Congo and Uganda (https://www.who.int/emergencies/disease-outbreak-news/item/2026-DON606) (accessed on 8 June 2026), as well as high cost and the requirement for cold-chain storage and delivery, which is particularly challenging in Africa. Thus, it is essential to develop more effective, affordable and easy-use drugs for combating this lethal viral disease.

**Figure 1:**
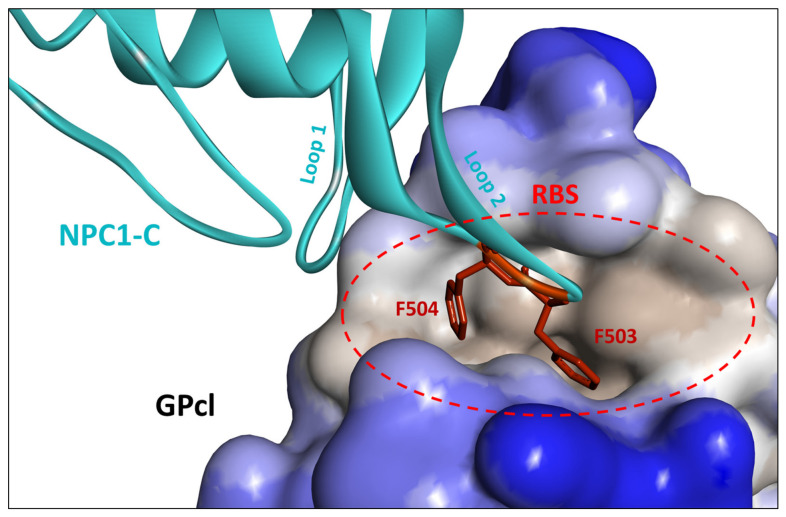
Interaction of NPC1 receptor domain C (NPC1-C) and cleaved EBOV GP (GPcl) (PDB 5F1B) during the viral entry at late endosomes or lysosomes. The recetor binding site (RBS) is indicated by the dashed red line and the two phenylalanine residues (F503 and F504) of NPC1 receptor are numbered based on the full-length sequence of NPC1 protein (Drawn by Discovery studio visualizer (BIOVIA, v21.1.0.)).

*Orthoebolavirus* is an RNA virus that has a negative-sense RNA genome (~19 kb) [6]. It infects cells through the macropinocytosis pathway by trafficking into early endosomes, late endosomes, and lysosomes for infection using the endosomal Niemann–Pick C1 (NPC1) protein as the receptor [7,8,9]. NPC1 is an intracellular cholesterol transporter, a large transmembrane protein with thirteen transmembrane helices, which consists of three domains: the N-terminal domain (Domain A), C-terminal domain (Domain I), and the middle domain (Domain C) [10,11]. NPC1 Domain C (NPC1-C) directly interacts with viral glycoprotein (GP), which is sufficient to support viral entry [12,13]. EBOV-GP is initially synthesized as a single polypeptide; then, it is cleaved by the host protease Furin into GP1 (outer surface part) and GP2 (transmembrane part) [14]. GP1 is a highly glycan shield, which consists of a receptor binding domain (RBD), a glycan cap, and a mucin-like domain (MLD) [15]. RBD is the key component for interacting with receptor NPC1-C, but the receptor binding site (RBS) is covered by the glycan cap and mucin-like domain. Thus, they must be removed by endosomal protease L&B cleavage to expose the receptor binding site. The cleaved GP is designed as GPcl, which can bind to the receptor NPC1-C for viral entry [12,13]. It is evident that the NPC1 receptor binding site (RBS) on GP is a principal target for drug development (Figure 1).

Therefore, we can utilize the interaction information between NPC1-C and viral GPcl to design peptide binders for binding to RBS and blocking viral entry. Several peptides designed based on the major contacts between loop 2 of NPC1-C and RBS of EBOV-GP have shown potent inhibition activities against filoviruses in vitro, and the data were published previously [16]. However, in this report, we have added the in vitro test against BDBV. BDBV was first identified in Uganda in 2007 [17], and it is well-known for causing severe EVD outbreaks in Africa, including the current deadly outbreak in central Africa. Herein, we report on the continuing in vivo studies of those leading peptide inhibitors in animal models against *Orthoebolavirus* and further explore the molecular mechanisms.

## 2. Materials and Methods

### 2.1. Peptides, Plasmids, Strains, and Cells

The designed peptides were synthesized from KE Biochem Co., LTD (Shanghai, China) or Pepmic Co., LTD (Suzhou, China). All purities of the synthesized peptides were at ≥95%, according to HPLC. The viral envelope glycoprotein genes were synthesized by GenScript (Piscataway, NJ, USA) as follows: EBOV (GenBank accession no. AIO11753.1) and BDBV (GenBank accession no. ACI28624). The plasmids of the A-MLV envelope (Amphotropic murine leukemia virus), HIV-1 backbone plasmid pSG3ΔEnv, and the TZM-bl cells were obtained from the NIH AIDS Reagent Program.

### 2.2. Pseudotyping Viruses

The HIV-1 backbone plasmid pSG3ΔEnv was used to create the pseudotyped viruses. The glycoprotein genes (GPs) of *Orthoebolavirus* (EBOV and BDBV) and A-MLV were cloned into the pCDNA3.1+ expression vector. Both plasmids of pSG3ΔEnv (6 µg) and the GP envelope (2 µg) were co-transfected into 293T cells in a 10 cm plate using transfection reagent polyethyleneimine (PEI) (24 µg). The plates were cultured in a tissue incubator at 37 °C and 5% CO_2_ for two days; then, the medium was harvested and centrifuged at 5000 rpm for 10 min to remove the cell debris. The supernatants containing the pseudotyped viruses were tiered using the TCID50-based method and stored at −80 °C in aliquots.

### 2.3. Inhibition Assay Against Pseudotyped Orthoebolavirus

The inhibition assay was performed following the adopted standard TZM-bl cell-based Luciferase assay protocol, as this cell line has a Luciferase report gene under the inducible promoter of Tat protein factor (Curr Protoc Immunol 2005) [18]. In brief, TZM-bl cells were set (6000 cells/well) in a 96-well plate for two days. The mixtures of viruses (Luciferase units ~50 K units/well, based on the optimization of infection) and protein samples were transferred onto the target cell wells for infection. One-day post infection, the media were removed, and the cells were washed once with PBS and incubated in fresh media for one more day. Then, the cells were lysed in 1× Passive Lysis Buffer (Promega, Madison, WI, USA) and kept at room temperature for 20 min. The Luciferase activity was measured using luciferin substrate (Promega) in a Veritas Luminometer. All the samples were assessed in triplicate and repeated once independently. The neutralization activities were calculated in comparison with the positive (virus only, 100% infectivity) and negative (cells only, 0% infectivity) controls. The IC_50_ values were calculated statistically using GraphPad Prism software (version 10.0.3).

### 2.4. Inhibition Assay Against Replication-Competent Orthoebolavirus

As previously described [16], HeLa cells were preincubated for 1 h with the peptides previously dissolved in water, before being challenged with wildtype *Orthoebolavirus zairense* (Zaire Mayinga EBOV) at an MOI of 0.1 to 0.3. After 36 h, the cells were fixed in formalin and removed from the BSL-4 lab for staining with a virus-specific monoclonal antibody against GP (4F3, IBT Bioservices, Rockville, MD, USA), followed by an Alexa 488-labeled anti-mouse secondary antibody (ThermoFisher, Waltham, MA, USA). The cell nuclei were stained using Hoechst 33342 (ThermoFisher) at 1:10,000. The wells were then imaged using an automated imaging microscope (Cytation 1, Biotek, Winooski, VT, USA). The images were processed using CellProfiler (Broad Institute, Cambridge, MA, USA) to count the infected and total cells using a custom pipeline that is available upon request. The infection efficiency was calculated as the ratio of infected cells to total cells [19]. Experiments were performed with at least 3 replicates.

### 2.5. Studies in Mice Models

#### 2.5.1. Ethics Statement

All the animal experiments conducted strictly followed the International Laboratory Animal Care and Use Committee’s approved protocols in compliance with the Animal Welfare Act PHS policy. The in vivo efficacy experiment in mice was conducted at the Texas Biomedical Research Institute (TBRI) and followed the International Laboratory Animal Care and Use Committee (IACUC) of TBRI and approved protocols TXBIO2018-007, IACUC #1648MU4, 29 January 2024. The in vivo pharmacokinetic (PK) studies in mice were conducted at the University of Nebraska Medical Center (UNMC) with the approved protocol IACUC #20-074-09-EP, 23 June 2023.

#### 2.5.2. In Vivo Pharmacokinetic (PK) Study

Eight uninfected 28–62-day-old female Balb/C mice (weight: ~25 g, *n* = 4, 2 groups) with jugular catheters were purchased from Charles River and were injected intravenously (slow infusion over 5 min) with doses of 25 mg/kg of body weight of *erp1* and *erp1c* peptides in PBS. Controls were not used, as pre-dose sampling was conducted on each animal. Doses for the mice were chosen based on previous experience with peptides and the principles of allometric scaling. Blood was collected from the animals pre- and post-injection at various time points (up to five post-injection plasma samples per day) via alternating cheek sampling and processed for plasma. Experimentation and sampling only lasted for 4 h given the half-life of the peptides. The samples were stored at −80 °C immediately until analysis via LC-MS/MS to determine the peptide concentrations. The plasma peptide levels were subjected to compartmental modeling (first-order elimination) and non-compartmental analysis with NONMEM version 7.4.3 (ICON PIc, Dublin, Ireland, and WinNonlin ver. 6.4 Certera Inc., Princeton, NJ, USA) to estimate the preliminary PK parameters. Covariates were not included for any of the compartmental PK modeling. All available animal data were included unless specified. All animals arrived at the UNMC animal facility and were allowed an appropriate standard acclimatization period prior to the initiation of the PK work.

#### 2.5.3. In Vivo Efficacy Test in Mice

Thirty-two Balb/c mice (7 weeks of age, 8 animals/group) in four groups (PBS mock group 9; Vehicle treated EBOV-only, group 10; peptide *m78abc* KE-163922 SC treated, group 11; and peptide *erp1c* KE-168824 SC treated, group 12) were chosen for the test from previous studies [16] (Details on animal testing design in Supplementary Materials S1). The mouse-adapted EBOV (MA EBOV) strain (1000 pfu) was administered via intraperitoneal (IP) injection. The peptide treatment was conducted about four hours post-infection with the subcutaneous (SC) injection of 200 µg/dose (~10 mg/kg) of peptide treatment, twice a day for 7 days. The mock control PBS group was a no-virus challenge. All animals were monitored and weighed daily. When moribund (or at the scheduled end-of-study, on day 21 post-challenge for the mock infection group), the animals were euthanized with CO_2_, and blood and tissue samples (liver, spleen, lung) were taken for histopathology analysis [20,21,22,23]. The histopathology report is included in the Supplementary Materials S1. At Texas Biomed, veterinary staff were blinded to group assignment until the finalization of post-life analysis. The animals were randomly assigned to each group; inclusion/exclusion criteria were based on age, sex, species, and weight. Animal husbandry and cage changing/cleaning were performed according to institutional SOPs. Animals were housed 8 per cage (per group); the caging complied with the cage requirements (type, floor space, height, etc.) of the Animal Welfare Act and the Guide for the Care and Use of Laboratory Animals. Rodent chow and enrichment were provided ad libitum and checked daily. Water from the Institutional Watering System was available. Animals were given nesting material and Shepherd Shacks. After virus exposure, mice were observed a minimum of twice daily. The endpoints in this study were survival/non-survival. Non-survival was defined by an animal having a terminal illness. Animal health was evaluated on a daily clinical observation score sheet. Euthanasia criteria were developed to minimize pain and suffering; a clinical score of 12 or higher indicated fulfillment of the euthanasia criteria, upon approval of the responsible veterinarian.

### 2.6. Molecular Docking

Molecular docking was performed using the Web-based program HPEPDOCK 2.0 (http://huanglab.phys.hust.edu.cn/hpepdock/data/6933276609c20, accessed on 8 June 2025) [24,25]. Flexible peptide-protein docking occurred through the fast modeling of peptide conformations and global/local sampling of binding orientations. The receptor binding domain (RBD) of EBOV-GP from PDB 5F1B chain A was used, and the specific contacting amino acids were set: W86, L111, and I170. In total, 1000 predictions for each peptide were produced, the 10 top-scored binding models were evaluated, and the best predictions were selected based on the docking scores and binding postures (see Supplementary Materials S3 for docking details).

### 2.7. Data Statistical Analysis

Graphpad Prism software (10.0.3) was used for all statistical analyses; to generate the neutralization figures; and to determine the average values, standard errors, and values of IC_50_ or CC_50_. One-way ANOVA, followed by Dunnett’s multiple comparisons test, was performed. Statistical analyses were performed with confidence intervals of 95%. In general, error bars were calculated from the means ± standard deviations (*n* = 3). For the in vivo PK work, the sample size was based on an estimated half-life of 1 h and a standard deviation of 0.2 h.

## 3. Results

### 3.1. Peptides erp1 and erp1c Inhibit Orthoebolavirus Infection In Vitro

**Figure 2:**
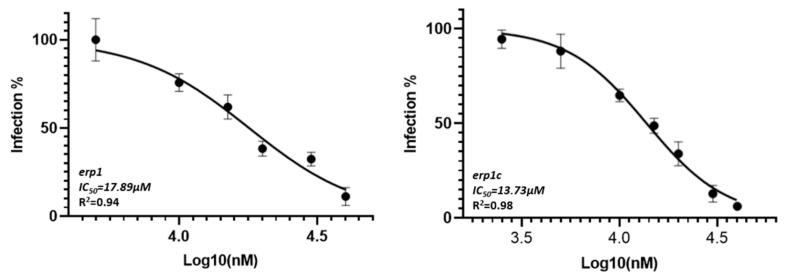
Inhibition assay of peptides *erp1* and *erp1c* against pseudotyped BDBV. Peptide *erp1c* is the cyclized form of *erp1*. The series of peptide concentrations were increased 2× from 2 µM to 32 µM, but the curves are in log10 scale. Each sample was evaluated in triplicate, and two independent experiments were conducted. TZM-bl cells were used for direct Luciferase activity readout in a Luminometer.

Peptides *erp1* (linear form) and *erp1c* (cyclized form) are a pair of inhibitors that were designed based on NPC1 receptor interaction with EBOV-GP. These two peptide inhibitors were previously observed to have strong activity against EBOV and MARV (*Orthomarburgvirus marburgense*, old name: *Marburgvirus marburgvirus*) infections in vitro [16]. Interestingly, the cyclized form (*erp1c*) exhibited more potent activity than its linear counterpart (*erp1*). In this report, we extended the use of these two inhibitors to another major *Orthoebolavirus* species, BDBV (*Orthoebolavirus bundibugyoense*). BDBV has 65.1% sequence identity with EBOV in GP. We pseudotyped the BDBV using HIV-1 as the backbone; we just used the BDBV GP gene as we pseudotyped EBOV previously (see Method section for more details). Then, we assessed these two peptides against the pseudotyped BDBV. The results indicated that both peptides *erp1* and *erp1c* inhibited BDBV infection with IC_50_ values of 17.89 µM and 13.73 µM, respectively (Figure 2). As noticed previously, the cyclized peptide also had better activity than its linear counterpart against EBOV.

### 3.2. Inhibition Against Replication-Competent EBOV Infection In Vitro

**Figure 3:**
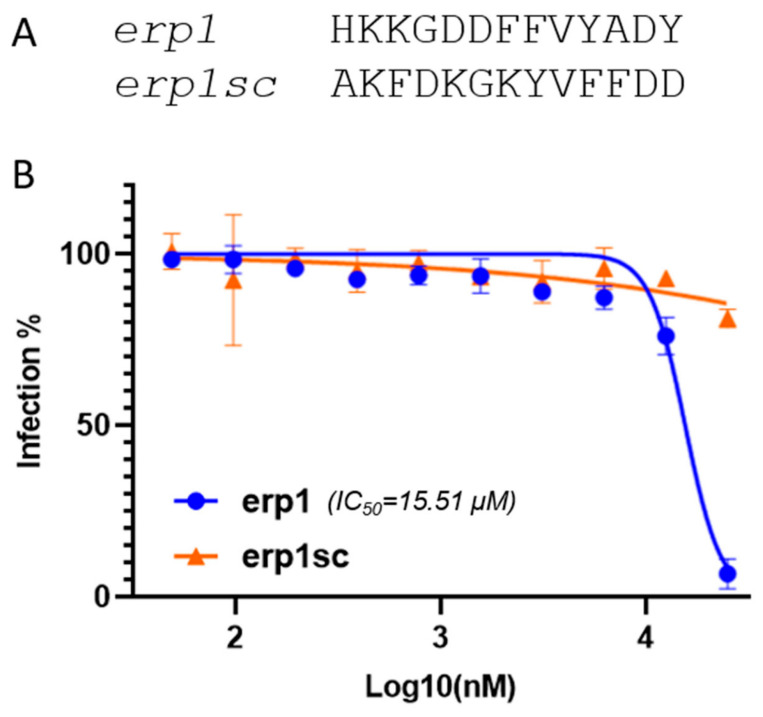
Inhibition assay of peptide *erp1* against replication-competent *Orthoebolavirus zairense* (EBOV) in vitro at BSL-4 containment. HeLa cells were used for the virus-neutralizing assay with the immunostaining method. (**A**). Peptide sequences of *erp1* and *erp1sc* (*erp1* sequence scramble peptide). (**B**). IC_50_ curves in log10 scale of *erp1* and *erp1sc*; each sample was assessed in triplicate (see the Materials and Methods for more details).

One representative peptide inhibitor *erp1* was submitted to the BSL-4 laboratory in the National Emerging Infectious Diseases Laboratories (NEIDL) for validation against wildtype (replication-competent) EBOV. The testing data were comparable with that of the pseudotyped virus with an IC_50_ value of 15.51 µM (Figure 3) [16]. The negative control peptide *erp1sc*, which is the *erp1* sequence scramble peptide, showed no inhibition activity. Hence, the *erp1* peptide can inhibit replication-competent EBOV infection, and the specific amino acid sequence order of the peptide is necessary for this function. This implies that the specific interactions between the peptide and receptor binding site of viral GP are required.

### 3.3. Specificity Analysis

**Figure 4:**
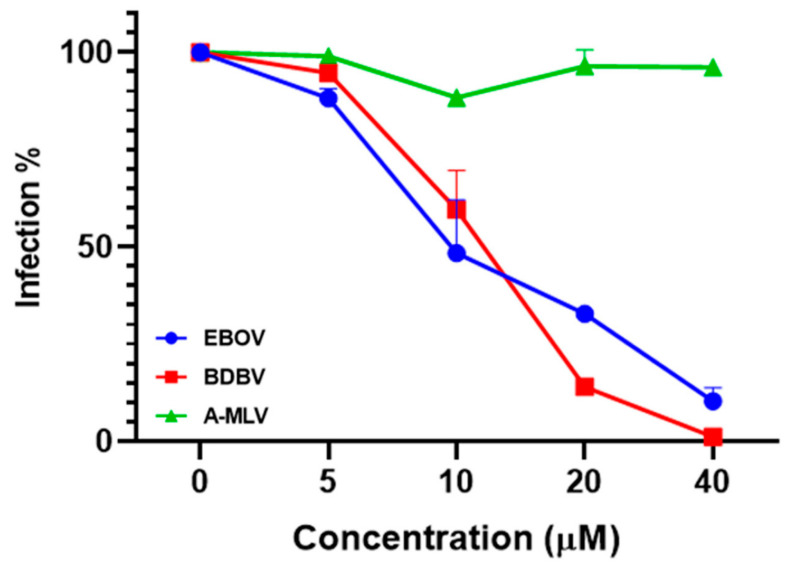
Specificity analysis of neutralization activity from the cyclized peptide *erp1c* for different viruses. The TZM-bl cell-based Luciferase assay was used for comparison and assessed side-by-side. A series of 2× concentrations (from 5 µM to 40 µM) of peptide *erp1c* were used for the test against EBOV (blue), BDBV (red), and A-MLV (green). Each sample was assessed in triplicate.

We used *erp1c* to assess the specificity of peptide inhibition. Amphotropic murine leukemia virus (A-MLV) was used as the negative control, as it is an RNA virus well-known for its ability to infect a wide variety of cells and is often used to study viral entry. Based on the side-by-side assay, peptide *erp1c* exhibited strong activity only against EBOV and BDBV with IC_50_ values of 11.6 µM and 11.3 µM, respectively, but no activity was observed against A-MLV (Figure 4). This suggests that the inhibition from the peptide *erp1c* is specific to *Orthoebolavirus* or filoviruses and not others, because this peptide inhibitor was designed by specifically targeting the NPC1 binding site.

### 3.4. Mutation Analysis of Peptide Inhibitors

**Figure 5:**
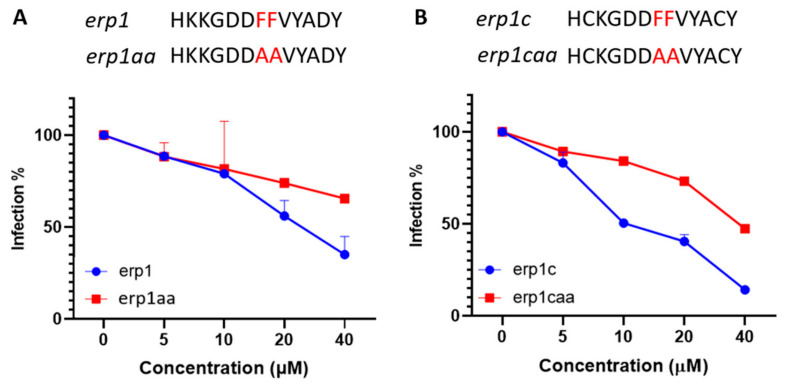
Inhibition analysis of peptide mutants against pseudotyped EBOV infection. (**A**). Wildtype linear peptide *erp1* (in blue) and mutant *erp1aa* (FF/AA) (in red). (**B**). Wildtype cyclized peptide *erp1c* (in blue) and mutant *erp1c* (FF/AA) (in red). The mutation sequences of FF/AA are in red. All peptides synthesized had a purity of ≥95%, according to HPLC certification data. The pseudotyped virus platform and TZM-bl cells were used for evaluations. Each sample was assessed in triplicate.

We demonstrated these two designed peptide inhibitors (*erp1* and *erp1c*) by targeting the receptor NPC1 binding site. Based on the hydrophobic interactions, the two phenylalanines (F7 and F8) in the peptide sequences presumably should play a critical role in the binding to the receptor binding site (RBS) on the GP for the inhibition of viral entry. To test this hypothesis, we mutated these two phenylalanines (FF) into two alanines (AA) in both forms of peptides *erp1* (linear) and *erp1c* (cyclized) for functional analysis. The data displayed that in both linear and cyclized peptides, the mutated FF/AA peptides clearly decreased the inhibition in a dose-dependent manner, in comparison with the counterpart wildtype peptide (Figure 5). The linear mutant peptide *erp1aa* increased the IC_50_ values from 25 µM to 109 µM with an estimated IC_50_ fold-change of 4.3; the cyclized mutant peptide *erp1caa* increased the IC_50_ values from 12.6 µM to 38.5 µM with an estimated IC_50_ fold-change of 3.0. These observed shifts in the inhibition curves strongly indicated that these two hydrophobic residues contribute significantly to the binding to the hydrophobic RBS.

### 3.5. Molecular Docking Analysis of Peptide Inhibitors

**Figure 6:**
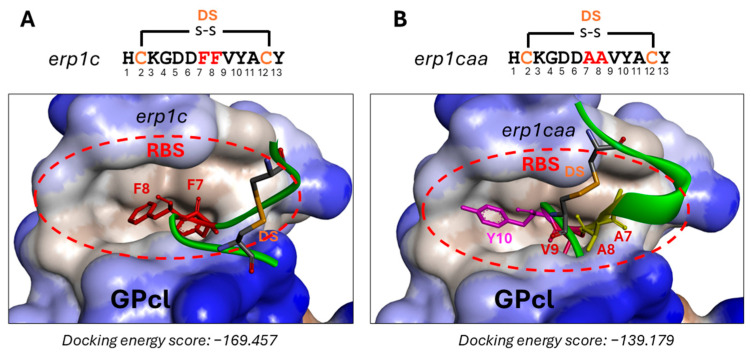
Molecular docking of peptides *erp1c* (**A**) and *erp1caa* (**B**) to the cleaved EBOV glycoprotein (GPcl). The web-based HPEPDOCK 2.0 docking program [24,25] was used for docking (see the Methods and Supplementary S3 for docking details). The peptide sequences are shown in the upper panel, where the two mutated amino acids F7F8/A7A8 are in red. DS represents the disulfide bond in purple; the peptide solid ribbon is in green; and the binding residues F7F8, A7A8, V9, and Y10 are colored sticks; The recetor binding site (RBS) is indicated by the dashed red line. (Drawn by Discovery studio visualizer (BIOVIA, v21.1.0.)).

The pair of cyclic peptides (*erp1c* wildtype and mutant *erp1caa* peptides) was analyzed via molecular docking. The results indicated that two phenylalanines (F7 and F8) were docked into the deep pockets of the receptor binding site as expected, where the binding mimics loop 2 of NPC1 (Figure 6). Interestingly, and unexpectedly, the mutant peptide *erp1caa* (FF/AA) adopted another way to achieve optimal binding by moving valine 9 (V9) and tyrosine 10 (Y10) to the deep pockets, rather than alanines (A7 and A8). Nonetheless, the mutant peptide showed a lower docking score than the wildtype peptide, which indicated the binding energy was significantly reduced. Thus, the two phenylalanines (FF) in the peptide sequence could play a significant role in binding to the RBS of GP to inhibit viral entry.

### 3.6. In Vivo PK Study of Peptide Inhibitors erp1 and erp1c

Four animals contributed levels for *erp1*, and only three animals contributed levels for *erp1c* (one animal was excluded due to processing issues). Each animal contributed approximately five blood samples, which were used to estimate the PK parameters. The concentration time profiles and complete preliminary in vivo PK analysis can be found in Supplementary Materials S2. Briefly, the *erp1* clearance (CL), volume of distribution (V), and half-life (T ½) were estimated to be 15.6 L/h (relative standard error [RSE]: 25%), 29.8 L (RSE: 36%), and 1.32 h (RSE: NA). For *erp1c*, the CL, Vd, and T ½ were estimated to be 3.85 L/h (RSE: 90%), 8.41 L (RSE: 38%), and 1.51 (RSE: NA) (for more detailed PK data, see Supplementary Materials S2). In general, we conclude that the cyclic peptide *erp1c* showed more variability than the linear *erp1*, but *erp1c* exhibited more stability than *erp1* in plasma.

### 3.7. Mice Model Study of Peptide Inhibitor erp1c

**Figure 7:**
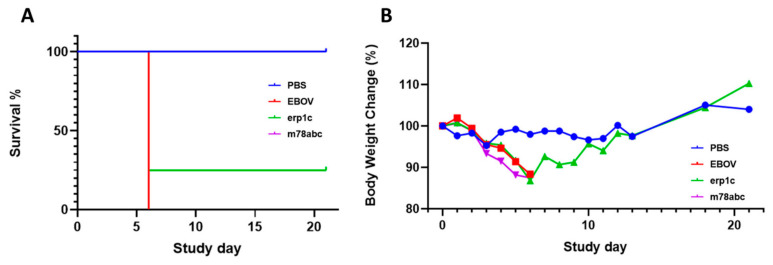
Mice model study of peptide *erp1c* challenged with replication-competent Ebola virus infection. The mice-adapted EBOV strain was used for the challenge infection of inbred Balb/c mice in four groups (8 mice/group) by intraperitoneal (IP) injection with a dose of 1000 pfu: PBS control (no EBOV infection) group in blue, EBOV infection control (no treatment) group in red, *erp1c*-treated group in green and *m78abc*-treated group in purple (see Methods section for more experimental details). (**A**). Animal survival curves at 7 days. The dose of peptides via subcutaneous (SC) injection was 200 µg/per animal (~10 mg/kg), twice a day for 7 days, but the total observation time was 21 days. Noted that the curves of the EBOV-infection control group (red) and *m78abc*-treated group (purple) overlapped. (**B**). Body weight loss curves over 21 days based on the average body weights of animal groups.

In vivo testing is important for the preclinical evaluation of antiviral inhibitors. The BSL-4 containment-based animal test was the most difficult and costly experiment in this project. Two cyclic peptide candidates, *erp1c* (KE-168824) and *m78abc* (KE-163922), were chosen for the mice model test, which is based on the in vitro efficacy determined from a previous study [16]. There were eight animals (Balb/c mice) per group, and the injection (SC, dose 200 µg) was administered twice a day for 7 days. The outcomes showed that in the no-treatment group (MA EBOV, red), all the mice died on day 6; the *m78abc*-treated group also died with no survivors (in purple). However, two out of eight mice survived in the *erp1c* peptide-treated group (*erp1c*, green), with a modest survival rate of 25% (25% vs. 0%). The negative control group (PBS Mock, blue) had no deaths (Figure 7A). Although statistical analysis would likely not achieve conventional statistical significance (*p* < 0.05) because of the small sample size and limited number of survivors, the two animals surviving from day 6 to day 21 (study endpoint and euthanized) are encouraging and important biologically from such lethal virus challenge. The observed partial protection suggests potential antiviral efficacy of the treatment and warrants further investigation in expanded studies. The histopathologic findings indicated that there were clear typical MA EBOV lesions in the liver and spleen from non-survivors, but there were no MA EBOV-like lesions and lymphoid hyperplasia in the two surviving animals. It is suggested that the deaths of animals were from MA EBOV infection (see Supplementary Materials S1). From the observation of the body weight changes, both the treated and not treated animals exhibited significantly reduced body weight from day 1 to day 6, but the treated group recovered some body weight; the control group (PBS Mock, blue) did not show much change in their body weight (Figure 7B). In conclusion, the animal challenge preliminary data suggests that the cyclized peptide *erp1c* likely provided partial protection against the MA EBOV virus infection.

## 4. Discussion

This in vivo study provided an example of using natural ligands such as receptors to design peptide binders targeting the viral receptor binding site (RBS), as reported in our previous in vitro study [16]. It demonstrated the advantages of ligand-based inhibitors because of their strong rationality and better specificity. In the case of the NPC1 receptor binding site (NPC1bs), the cyclic peptide *erp1c* showed better activity than the linear counterpart peptide *erp1*, as the cyclic *erp1c* mimics loop 2 binding of the nature ligand NPC1 receptor (Domain C). Presumably, NPC1-based inhibitors have the potential for a broad coverage of filoviruses. Indeed, the inhibitors *erp1* and *erp1c* showed activity against two different genera of *Orthoebolavirus* and *Orthomaburgervirus* from this study and a previous study [16] because they share the same receptor (NPC1) for viral entry, although the binding domain (RBD) sequence is only 48% identity.

The mouse model study remains relatively preliminary, reflecting the limited information currently available in this field. Although the observed protective efficacy was modest, the results are both encouraging and informative. Notably, in studies involving highly lethal challenge models, increasing the sample size would be important to enable robust statistical analysis. In addition, the administered dose (~10 mg/kg) appears to be suboptimal given that the IC_50_ values of these peptides fall within the low micromolar range; therefore, dose optimization will be necessary in future studies to improve efficacy. We also acknowledge limitations of our in vivo PK analysis and caution against overinterpreting the clinical implications currently. These studies were conducted as a preliminary, exploratory analysis. The study was designed as a pilot to characterize preliminary PK properties and variability and was not intended to support formal PK/PD or exposure–response conclusions. Future work will incorporate comprehensive PK/PD analyses, including evaluation of exposure relative to IC_50_ values and linkage to antiviral outcomes.

Based on the IC_50_ data, peptide *m78abc* exhibited an IC_50_ of 16.1 µM against pseudotyped EBOV and failed to confer protection in vivo, as all animals succumbed to infection. In contrast, peptide *erp1c* demonstrated IC_50_ values of 12.3 µM against pseudotyped EBOV and 10.5 µM against wildtype EBOV in prior in vitro studies [16] and showed partial protection with modest efficacy in vivo. These findings suggest that enhancing inhibitory potency and further reducing IC_50_ values will be critical for achieving improved therapeutic efficacy in vivo.

Nevertheless, improving the inhibitor design to increase the potency and reduce the IC_50_ concentrations will be essential for the in vivo efficacy at the same dosing. This can be through modifying the peptide sequences to increase the binding affinity with the receptor binding site via structure-based design. Other modifications to improve the peptide properties for targeting are also important. For example, since this receptor is located in endosomes or lysosomes, increasing the properties for membrane penetration or internalization that would be beneficial for the function of peptide-based inhibitors. Therefore, one could consider adding cell membrane-penetrating peptide sequences (CPPs) to increase the cell membrane permeability [26,27,28] or adding phosphatidylserine (PS) to the peptides to bind to cell surface molecules, such as Tim-1, to enhance peptide trafficking through the macropinocytosis pathway to enter endosomes/lysosomes [29,30,31].

Peptide-based drugs have unique properties in comparison to small molecule- or protein-based drugs, such as a lower toxicity, higher specificity, and better flexibility; therefore, they have become increasingly valuable for treating human diseases [32,33,34]. For Ebola viral disease (EVD) or other filoviral diseases such as Marburg virus disease (MVD), peptide-based drugs to target the receptor binding site would be especially significant, because the filoviruses share the same receptor NPC1 for infection. Hence, they can share the same inhibitors to block viral entry. By using this approach, we may be able to identify shared peptide-based drugs for all filoviruses. In conclusion, in this study, we have demonstrated with in vitro and in vivo data that a receptor-based peptide design to target the receptor binding site could be an effective way to block filovirus infection. The cyclized peptide (*erp1c*) exhibits a promising direction for the further development of peptide-based drugs in filovirus infections.

## Data Availability

The data presented in this study are available upon request from the corresponding author.

## Supplementary Materials

The following supporting information can be downloaded at: https://www.mdpi.com/article/10.3390/pathogens15060640/s1, S1, Summary Pathological report; S2, Pharmacokinetic study report; S3, Input information for docking using HPEPDOCK 2.0.

## Abbreviations

The following abbreviations are used in this manuscript:

EBOV	*Orthoebolavirus zairense*
BDBV	*Orthoebolavirus bundibugyoense*
SUDV	*Orthoebolavirus sudanense*
RESTV	*Orthoebolavirus restone**nse*
TAFV	*Orthoebolavirus taiense*
BOMV	*Orthoebolavirus bombaliense*
MARV	*Orthomarburgvirus marburgense*
A-MLV	Amphotropic murine leukemia virus
EVD	Ebola virus disease
MVD	Marburg virus disease
NPC1	Niemann–Pick disease type C1
NPC1-C	NPC1 Domain C
GPcl	Cleaved GP

## References

[1] Biedenkopf N., Bukreyev A., Chandran K., Di Paola N., Formenty P.B.H., Griffiths A., Hume A.J., Muhlberger E., Netesov S.V., Palacios G. (2023). Renaming of genera Ebolavirus and Marburgvirus to Orthoebolavirus and Orthomarburgvirus, respectively, and introduction of binomial species names within family Filoviridae. Arch. Virol..

[2] Biedenkopf N., Bukreyev A., Chandran K., Di Paola N., Formenty P.B.H., Griffiths A., Hume A.J., Muhlberger E., Netesov S.V., Palacios G. (2024). ICTV Virus Taxonomy Profile: Filoviridae 2024. J. Gen. Virol..

[3] Ohimain E.I., Silas-Olu D. (2021). The 2013-2016 Ebola virus disease outbreak in West Africa. Curr. Opin. Pharmacol..

[4] Diakou K.I., Mitsis T., Pierouli K., Papakonstantinou E., Bongcam-Rudloff E., Wayengera M., Vlachakis D. (2021). Ebola Virus Disease and Current Therapeutic Strategies: A Review. Adv. Exp. Med. Biol..

[5] Tshiani Mbaya O., Mukumbayi P., Mulangu S. (2021). Review: Insights on Current FDA-Approved Monoclonal Antibodies Against Ebola Virus Infection. Front Immunol..

[6] Bodmer B.S., Hoenen T., Wendt L. (2024). Molecular insights into the Ebola virus life cycle. Nat. Microbiol..

[7] Carette J.E., Raaben M., Wong A.C., Herbert A.S., Obernosterer G., Mulherkar N., Kuehne A.I., Kranzusch P.J., Griffin A.M., Ruthel G. (2011). Ebola virus entry requires the cholesterol transporter Niemann-Pick C1. Nature.

[8] Cote M., Misasi J., Ren T., Bruchez A., Lee K., Filone C.M., Hensley L., Li Q., Ory D., Chandran K. (2011). Small molecule inhibitors reveal Niemann-Pick C1 is essential for Ebola virus infection. Nature.

[9] Saeed M.F., Kolokoltsov A.A., Albrecht T., Davey R.A. (2010). Cellular entry of ebola virus involves uptake by a macropinocytosis-like mechanism and subsequent trafficking through early and late endosomes. PLoS Pathog..

[10] Zhao Y., Ren J., Harlos K., Stuart D.I. (2016). Structure of glycosylated NPC1 luminal domain C reveals insights into NPC2 and Ebola virus interactions. FEBS Lett..

[11] Davies J.P., Ioannou Y.A. (2000). Topological analysis of Niemann-Pick C1 protein reveals that the membrane orientation of the putative sterol-sensing domain is identical to those of 3-hydroxy-3-methylglutaryl-CoA reductase and sterol regulatory element binding protein cleavage-activating protein. J. Biol. Chem..

[12] Gong X., Qian H., Zhou X., Wu J., Wan T., Cao P., Huang W., Zhao X., Wang X., Wang P. (2016). Structural Insights into the Niemann-Pick C1 (NPC1)-Mediated Cholesterol Transfer and Ebola Infection. Cell.

[13] Wang H., Shi Y., Song J., Qi J., Lu G., Yan J., Gao G.F. (2016). Ebola Viral Glycoprotein Bound to Its Endosomal Receptor Niemann-Pick C1. Cell.

[14] Volchkov V.E., Feldmann H., Volchkova V.A., Klenk H.D. (1998). Processing of the Ebola virus glycoprotein by the proprotein convertase furin. Proc. Natl. Acad. Sci. USA.

[15] Lee J.E., Fusco M.L., Hessell A.J., Oswald W.B., Burton D.R., Saphire E.O. (2008). Structure of the Ebola virus glycoprotein bound to an antibody from a human survivor. Nature.

[16] Wang L.L., Estrada L., Wiggins J., Anantpadma M., Patten J.J., Davey R.A., Xiang S.H. (2022). Ligand-based design of peptide entry inhibitors targeting the endosomal receptor binding site of filoviruses. Antivir. Res..

[17] Towner J.S., Sealy T.K., Khristova M.L., Albarino C.G., Conlan S., Reeder S.A., Quan P.L., Lipkin W.I., Downing R., Tappero J.W. (2008). Newly discovered ebola virus associated with hemorrhagic fever outbreak in Uganda. PLoS Pathog..

[18] Montefiori D.C. (2005). Evaluating neutralizing antibodies against HIV, SIV, and SHIV in luciferase reporter gene assays. Curr. Protoc. Immunol..

[19] Keiser P.T., Anantpadma M., Staples H., Carrion R., Davey R.A. (2021). Automation of Infectious Focus Assay for Determination of Filovirus Titers and Direct Comparison to Plaque and TCID(50) Assays. Microorganisms.

[20] Bray M., Davis K., Geisbert T., Schmaljohn C., Huggins J. (1998). A mouse model for evaluation of prophylaxis and therapy of Ebola hemorrhagic fever. J. Infect. Dis..

[21] Gibb T.R., Bray M., Geisbert T.W., Steele K.E., Kell W.M., Davis K.J., Jaax N.K. (2001). Pathogenesis of experimental Ebola Zaire virus infection in BALB/c mice. J. Comp. Pathol..

[22] Wang L.L., Alfson K., Eaton B., Mattix M.E., Goez-Gazi Y., Holbrook M.R., Carrion R., Xiang S.H. (2025). Algal Lectin Griffithsin Inhibits Ebola Virus Infection. Molecules.

[23] Pessi A., Bixler S.L., Soloveva V., Radoshitzky S., Retterer C., Kenny T., Zamani R., Gomba G., Gharabeih D., Wells J. (2019). Cholesterol-conjugated stapled peptides inhibit Ebola and Marburg viruses in vitro and in vivo. Antivir. Res..

[24] Zhou P., Jin B., Li H., Huang S.Y. (2018). HPEPDOCK: A web server for blind peptide-protein docking based on a hierarchical algorithm. Nucleic Acids Res..

[25] Tao H., Zhao X., Zhang K., Lin P., Huang S.Y. (2022). Docking cyclic peptides formed by a disulfide bond through a hierarchical strategy. Bioinformatics.

[26] Gori A., Lodigiani G., Colombarolli S.G., Bergamaschi G. (2023). Vitali A: Cell Penetrating Peptides: Classification, Mechanisms, Methods of Study, and Applications. ChemMedChem.

[27] Li G., Su B., Fu P., Bai Y., Ding G., Li D., Wang J., Yang G., Chu B. (2022). NPC1-regulated dynamic of clathrin-coated pits is essential for viral entry. Sci. China Life Sci..

[28] Zorko M., Langel U. (2022). Cell-Penetrating Peptides. Methods Mol. Biol..

[29] Shah S.S., Casanova N., Antuono G., Sabatino D. (2020). Polyamide Backbone Modified Cell Targeting and Penetrating Peptides in Cancer Detection and Treatment. Front Chem..

[30] Jankowski A.M., Ensign M.A., Maisel K. (2025). Cell-penetrating peptides as facilitators of cargo-specific nanocarrier-based drug delivery. Nanoscale.

[31] Moller-Tank S., Kondratowicz A.S., Davey R.A., Rennert P.D., Maury W. (2013). Role of the phosphatidylserine receptor TIM-1 in enveloped-virus entry. J. Virol..

[32] Wang L., Wang N., Zhang W., Cheng X., Yan Z., Shao G., Wang X., Wang R., Fu C. (2022). Therapeutic peptides: Current applications and future directions. Signal Transduct. Target Ther..

[33] Xiao W., Jiang W., Chen Z., Huang Y., Mao J., Zheng W., Hu Y., Shi J. (2025). Advance in peptide-based drug development: Delivery platforms, therapeutics and vaccines. Signal Transduct. Target Ther..

[34] Warthen J.L., Lueckheide M.J. (2024). Peptides as Targeting Agents and Therapeutics: A Brief Overview. Biomacromolecules.

