# Supplementary material for: Development of Peptide Entry Inhibitors Targeting the Endosomal Receptor NPC1 Binding Site of Orthoebolavirus
Source: Pathogens. 2026 Jun 16;15(6):640. doi: 10.3390/pathogens15060640 (PMC13304712; doi:10.3390/pathogens15060640)
Supplement: Supplementary file 1 [file pathogens-15-00640-s001.zip › pathogens-4270772-supplementary.pdf]

S1

SUMMARY PATHOLOGY REPORT

1648 MU
(Peptide groups only)

Efficacy of Novel Ebola virus Countermeasures in Mice

Study Director: Ricardo Carrion Jr., PhD
Texas Biomedical Research Institute

Study Pathologist: Marc E. Mattix, DVM, MSS
Diplomate, ACVP
Nonclinical Pathology Services, LLC

COMPILED FOR:
Texas Biomedical Research Institute
8715 W. Military Dr.
San Antonio, TX 78227-5302

TABLE OF CONTENTS

**INTRODUCTION**	**Page 3**
**Study Title**	**3**
**Purpose**	**3**
**MATERIALS AND METHODS**	**3**
**PATHOLOGY RESULTS**	**3-6**
**Survival**	**3**
**Histologic findings**	**4-5**
**Group 9**	**4**
**Group 10**	**4**
**Group 11**	**5**
**Group 12**	**5**
**CONCLUSION**	**6**
**REFERENCES**	**6**
**REPORT SUBMISSION**	**6**

INTRODUCTION

Study Title: Efficacy of novel Ebola virus countermeasures in mice.

Purpose: The purpose of this study is to evaluate the efficacy of novel EBOV countermeasures in mice.

MATERIALS AND METHODS

Study Site: Texas Biomedical Research Institute
Animals: Mice
Challenge Agent: Mouse adapted (MA) EBOV, 1000 PFU IP
Test Articles: KE-163922; KE-168824
Vehicle: PBS

Experimental Study: Forty mice were assigned to 5 groups consisting of 8 mice per group, as detailed in Table S1.

Table S1. Experimental Design

Group No.	No. Animals	Experimjental Objective	Treatment SC	Challenge Agent
9	8	PBS Mock; vehicle treated, unexposed	N/A	N/A
10	8	Vehicle treated, MA EBOV exposed	N/A	MA EBOV
11	8	KE-163922 SC	SD 0,9 (SID)<br>SD1-8 (BID)	MA EBOV
12	8	KE-168824 SC	SD 0,9 (SID)<br>SD1-8 (BID)	MA EBOV

N/A – Not applicable.

Gross Necropsy:
Necropsies were conducted in accordance with Texas Biomed SOP 903. Tissue samples (liver, spleen, and lung) were collected for microscopic evaluation.

Histopathology:
Tissues were fixed by immersion in 10% neutral-buffered formalin for a minimum of fourteen days, then trimmed, routinely processed, and embedded in paraffin. Sections of the paraffin-embedded tissues were cut at 5 μm thick, and histology slides were deparaffinized, stained with hematoxylin and eosin (H&E), coverslipped, and labeled. Slides were evaluated by a board-certified veterinary pathologist using a light microscope. The results of histopathological examination of each animal were summarized in the Summary Pathology Report by the Study Pathologist (Table S2 to Table S5).

PATHOLOGY RESULTS

Survival
All PBS unexposed control animals survived to the Day 21 terminal euthanasia. Two of 8 animals assigned to the KE-168824 group (Group 12) survived to the terminal euthanasia. All vehicle treated MA EBOV exposed mice (Group 2) and remaining test article-treated MA EBOV-exposed mice (Group 11 and 12) died or were euthanized prior to the end of protocol, between Day 3 and 7.

GROUP 9 (PBS mock; vehicle treated, unexposed)

Histologic findings

No significant microscopic findings were noted. Mononuclear cell infiltration within the liver, rarely surrounding apoptotic hepatocytes, was considered spontaneous change unrelated to experimental manipulation. Findings are summarized in Table S2.

Table S2. Summary of select Group 9 histopathology findings

	Accession No.	24-0100							
	Study Day	21	21	21	21	21	21	21	21
	Animal No.	A*	B*	C*	D*	E*	F*	G*	H*
LIVER	Hepatocellular necrosis	0	0	0	0	0	0	0	0
	Inflammation	0	0	0	0	0	0	0	0
	Hepatocellular vacuolation	0	0	0	0	0	0	0	0
	Cytoplasmic inclusions	0	0	0	0	0	0	0	0
	Mononuclear cell infiltration	0	1	1	0	0	0	0	0
SPLEEN	Lymphoid depletion	0	0	0	0	0	0	0	0
	Lymphocytolysis	0	0	0	0	0	0	0	0
	Fibrin	0	0	0	0	0	0	0	0
	Necrosis, focal	0	0	0	0	0	0	0	0
	Lymphoid hyperplasia	0	0	0	0	0	0	0	0
LUNG	Inflammation	0	0	0	0	0	0	0	0
	Increased alveolar macrophages	0	0	0	0	0	0	0	0

1- Minimal; 2- Mild; 3- Moderate; 4- Marked; 5- Severe; P - Ungraded finding present; 0 - Finding not present; NE - Not evaluated; * - Survivor

Group 10 (Vehicle treated, MA EBOV exposed)

Histologic findings

Microscopic changes consistent with EBOV infection consisted of hepatocellular necrosis with mixed cell inflammation and intracytoplasmic inclusion bodies and splenic lymphoid depletion with lymphocytolysis. Fibrin deposition was absent in tissues evaluated. Findings are summarized in Table S3.

Table S3. Summary of select Group 10 histopathology findings

	Accession No.	24-0101							
	Study Day	6	6	6	6	6	6	6	6
	Animal No.	A	B	C	D	E	F	G	H
LIVER	Hepatocellular necrosis	3	3	4	4	2	4	3	4
	Inflammation	2	3	2	3	1	2	2	2
	Hepatocellular vacuolation	0	1	0	1	2	0	0	0
	Cytoplasmic inclusions	P	P	P	P	0	P	P	P
	Mononuclear cell infiltration	0	0	0	0	0	0	0	0
SPLEEN	Lymphoid depletion	3	3	2	2	3	3	3	3
	Lymphocytolysis	3	3	2	2	3	3	2	3
	Fibrin	0	0	0	0	0	0	0	0
	Necrosis, focal	0	0	0	0	0	0	0	0
	Lymphoid hyperplasia	0	0	0	0	0	0	0	0
LUNG	Inflammation	0	0	0	0	0	0	0	0
	Increased alveolar macrophages	0	0	0	0	0	0	0	0

1- Minimal; 2- Mild; 3- Moderate; 4- Marked; 5- Severe; P - Ungraded finding present; 0 - Finding not present; NE - Not evaluated

GROUP 11 (KE-163922)

Histologic findings

Microscopic changes consistent with EBOV infection consisted of hepatocellular necrosis with mixed cell inflammation and intracytoplasmic inclusion bodies and splenic lymphoid depletion with

lymphocytolysis. Fibrin deposition was not noted in tissues evaluated. EBOV-related findings were similar in character to those noted in the positive control group. One consistent finding within the liver, hepatocellular vacuolation, was not noted in the control groups, suggesting a test article-related effect. The finding was characterized by enlarged hepatocytes containing numerous small clear cytoplasmic vacuoles with discrete, well-defined margins (microvesicular vacuolation). Findings are summarized in Table S4.

Table S4. Summary of select Group 11 histopathology findings

	Accession No.	24-0102							
	Study Day	6	6	6	6	6	6	6	6
	Animal No.	A	B	C	D	E	F	G	H
LIVER	Hepatocellular necrosis	3	2	3	3	3	3	1	3
	Inflammation	2	1	3	2	2	2	1	2
	Hepatocellular vacuolation	1	1	1	0	0	2	2	0
	Cytoplasmic inclusions	P	P	P	P	P	P	0	P
	Mononuclear cell infiltration	0	0	0	0	0	0	0	0
SPLEEN	Lymphoid depletion	2	1	2	2	3	3	2	2
	Lymphocytolysis	2	1	2	1	2	1	2	2
	Fibrin	0	0	0	0	0	0	0	0
	Necrosis, focal	0	0	0	0	0	0	0	0
	Lymphoid hyperplasia	0	0	0	0	0	0	0	0
LUNG	Inflammation	0	0	0	0	0	0	0	0
	Increased alveolar macrophages	0	0	0	0	0	0	0	0

1- Minimal; 2- Mild; 3- Moderate; 4- Marked; 5- Severe; P - Ungraded finding present; 0 - Finding not present

GROUP 12 (KE-168824)

<u>Histologic findings</u>

Microscopic changes consistent with EBOV infection consisted of hepatocellular necrosis with mixed cell inflammation and intracytoplasmic inclusion bodies and splenic lymphoid depletion with lymphocytolysis. EBOV-related findings were similar in character to those noted in the positive control group. One finding noted within the liver of unscheduled death mice only, hepatocellular vacuolation, was not noted in the control groups nor within the surviving 2 mice in this group. The finding was characterized by enlarged hepatocytes that contained numerous small clear cytoplasmic vacuoles with discrete, well-defined margins (microvesicular vacuolation). Findings are summarized in Table S4. No EBOV-related findings were noted in the surviving mice. Findings are summarized in Table S5.

Table S5. Summary of select Group 12 histopathology findings

	Accession No.	24-0103							
	Study Day	6	6	6	6	6	21	6	21
	Animal No.	A	B	C	D	E	F*	G	H*
LIVER	Hepatocellular necrosis	3	2	4	1	3	0	4	0
	Inflammation	2	2	1	1	2	0	2	0
	Hepatocellular vacuolation	2	2	2	2	2	0	2	0
	Cytoplasmic inclusions	P	P	P	0	P	0	P	0
	Mononuclear cell infiltration	0	0	0	0	0	1	0	0
SPLEEN	Lymphoid depletion	2	2	3	1	2	0	2	0
	Lymphocytolysis	2	2	2	2	2	0	2	0
	Fibrin	0	0	0	0	0	0	0	0
	Necrosis, focal	0	0	0	0	0	0	0	0
	Lymphoid hyperplasia	0	0	0	0	0	3	0	3
LUNG	Inflammation	0	0	0	0	0	0	0	0
	Increased alveolar macrophages	0	0	0	0	0	0	0	0

1- Minimal; 2- Mild; 3- Moderate; 4- Marked; 5- Severe; P - Ungraded finding present; 0 - Finding not present; NE - Not evaluated; * - Survivor

CONCLUSION

Forty mice were assigned to 8 groups, as described in Table S1. All mock exposed mice survived to the terminal euthanasia. All vehicle treated mice exposed to MA EBOV died or were euthanized on Day 6. All KE-163922-treated mice died or were euthanized on day 6 with microscopic findings similar to those noted in the vehicle MA EBOV-exposed group. Six of 8 KE-168824-treated mice died or were euthanized on Day 6 with microscopic findings similar to the MA EBOV control group. Two KE-168824-treated mice survived to the terminal euthanasia. Neither surviving mouse had microscopic findings suggestive of active EBOV infection. Both mice had moderate lymphoid hyperplasia in the spleen, considered to be a treatment-related effect.

MA EBOV-related microscopic findings in EBOV-exposed, unscheduled death control and treated mice included hepatocellular necrosis with inflammation and intracytoplasmic inclusions and splenic lymphoid depletion with lymphocytolysis. Fibrin deposition was not noted, a unique feature of MA EBOV infection in mice [21]. Hepatocellular vacuolation, characterized by multiple small cytoplasmic vacuoles with distinct borders, was noted in the unscheduled death mice from each of the treated groups. The finding was consistent with microvascular vacuolation and has been described as a MA EBOV-related finding in mice [20].

REFERENCES

20. Bray, M.; Davis, K.; Geisbert, T.; Schmaljohn, C.; Huggins, J. A mouse model for evaluation of prophylaxis and therapy of Ebola hemorrhagic fever. J. Infect. Dis. 1998, 178, 651–661. [Google Scholar] [CrossRef] [PubMed]

21. Gibb, T.R.; Bray, M.; Geisbert, T.W.; Steele, K.E.; Kell, W.M.; Davis, K.J.; Jaax, N.K. Pathogenesis of experimental Ebola Zaire virus infection in BALB/c mice. J. Comp. Pathol. 2001, 125, 233–242. [Google Scholar] [CrossRef]

REPORT SUBMISSION

Texas Biomed 1648 MU Pathology Report

Report Submitted By:

______________________________ ______________

Marc E. Mattix, DVM, MSS Date

Diplomate, ACVP

Study Pathologist

S2

Pharmacokinetic study report

Figure S1

Peptide 1 (erp1) (IV Catheter)

CL (RSE) L/h	V (RSE) L	T ½ (hrs)	Ke (hr^{-1})
15.6(25%)	29.8 (36%)	1.32	0.5234

1-compartmental population PK analysis

ID	Tmax (h)	Cmax (ug/L)	AUCall h*ug/L
20	0.25	16.2	26.657
21	1.0833	29.9	33.940
22	0.65	2.69	3.147
23	0.2	25.2	29.36

NCA

Figure S2 Individual mouse dose response to petide 1(erp1)

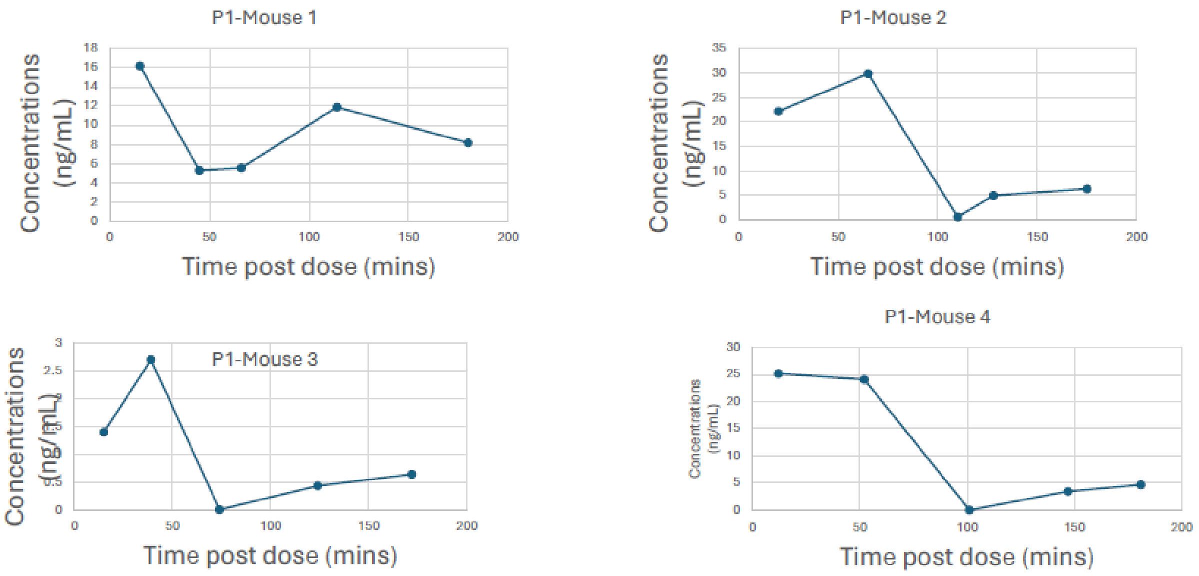

Figure S3

Peptide 2 (erp1c) (IV Catheter)

CL (RSE) L/h	V (RSE) L	IIV~CL(RSE)	T ½ (hrs)	Ke (hr^{-1})
3.85(90%)	8.41 (38%)	1.65(82%)	1.51	0.46

1-compartmental population PK analysis

ID	Tmax (h)	Cmax (ug/L)	AUCall h*ug/L
15	0.5	130	236.1339
16	0.0833	12.8	14.505
18	1.667	12.6	31.93
19	1.0833	34	80.055

NCA

Figure S4 Individual mouse dose response to peptide 2 (erp1c)

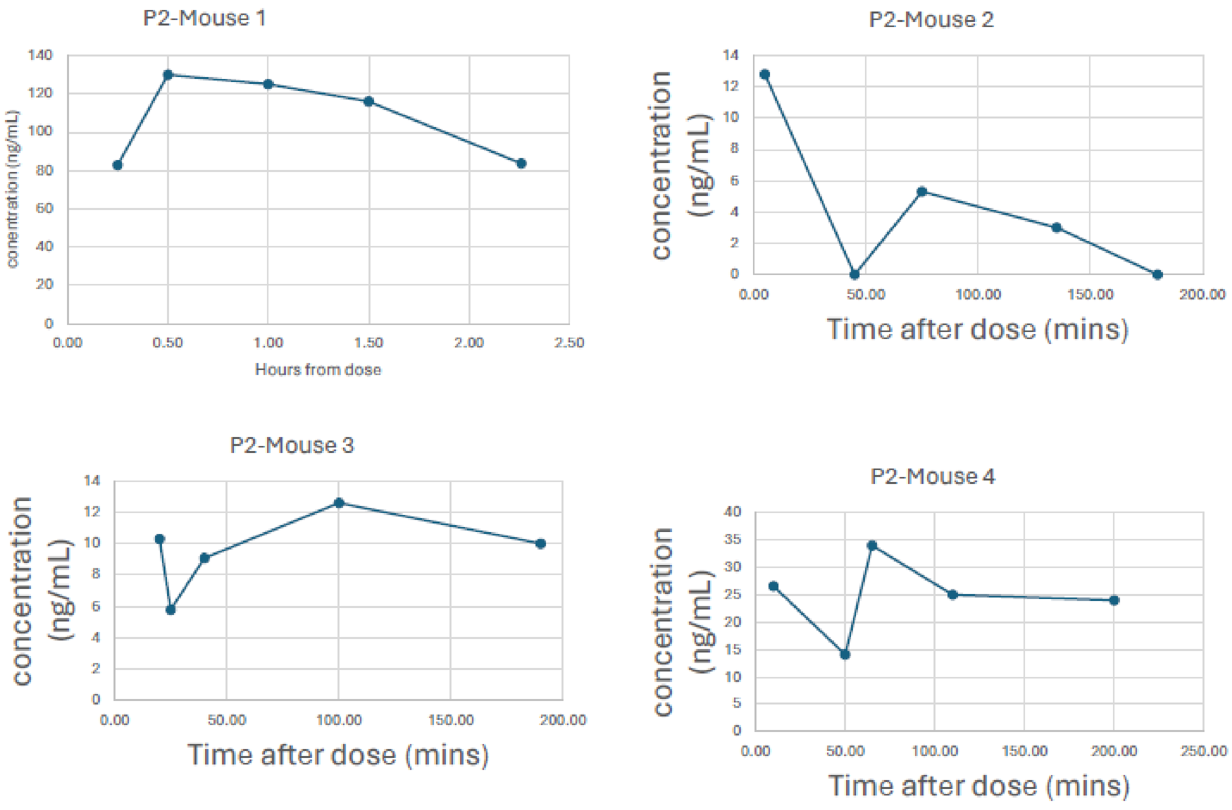

S3

Input information for docking using HPEPDOCK 2.0

erp1c

```
Receptor PDB file:
Receptor PDB ID: 5F1B:A
Receptor sequence:

Receptor sequence file:

Peptide PDB file:
Peptide PDB ID:
Peptide sequence:
HCKGDDFFVYACY -ss 2 12
Peptide sequence file:

Docking mode: dock
Binding site reference file:
Binding site residues: 86:A, 111:A, 170:A,
Number of peptide conformations: 1000
Number of output binding modes:
Rigid peptide docking:

Email address: sxiang2@unl.edu
Job name: erp1c
```

erp1caa

```
Receptor PDB file:
Receptor PDB ID: 5F1B:A
Receptor sequence:

Receptor sequence file:

Peptide PDB file:
Peptide PDB ID:
Peptide sequence:
HCKGDDAAVYACY -ss 2 12
Peptide sequence file:

Docking mode: dock
Binding site reference file:
Binding site residues: 86:A, 111:A, 170:A,
Number of peptide conformations: 1000
Number of output binding modes:
Rigid peptide docking:

Email address: sxiang2@unl.edu
Job name: erp1caa
```
